# Supplementary material for: A Metabolomic Approach to Unexplained Syncope
Source: Biomedicines. 2024 Nov 19;12(11):2641. doi: 10.3390/biomedicines12112641 (PMC11591916; doi:10.3390/biomedicines12112641)
Supplement: Supplementary file 1 [file biomedicines-12-02641-s001.zip › biomedicines-3267874-supplementary.pdf]

A metabolomic approach to unexplained syncope.

Susanna Longo[1], Ilaria Cicalini[2,3], Damiana Pieragostino[2,3], Vincenzo De Laurenzi[2,3], Jacopo M. Legramante[1], Rossella Menghini[1], Stefano Rizza[1], Massimo Federici[1]

susanna.longo@uniroma2.it,ilaria.cicalini@unich.it,damiana.pieragostino@unich.it, vincenzo.delaurenzi@unich.it legraman@uniroma2.it, menghini@uniroma2.it, rizza@med.uniroma2.it, federicm@uniroma2.it.

[1]Department of Systems Medicine, University of Rome Tor Vergata
[2]Department of Innovative Technologies in Medicine and Dentistry, "G. d'Annunzio" University of Chieti-Pescara, 66100 Chieti, Italy.
[3]Center for Advanced Studies and Technology (CAST), "G. d'Annunzio" University of Chieti-Pescara, 66100 Chieti, Italy.

Address correspondence to:
e-mail federicm@uniroma2.it Department of Systems Medicine University of Rome Tor Vergata, Via Montpellier 1, 00133 Rome ITALY

Table S1: Electrospray ionization mass spectrometry (ESI-MS) acquisition parameters used for the analysis of amino acids (AAs), free carnitine (C0), acylcarnitines (ACCs), ketones as succinylacetone (SA), nucleosides and lysophospholipids. Table S1 shows the MS/MS transitions for each analysed metabolites and the corresponding internal standard (IS, shown in bold) used for quantitative analysis.

Abbreviation Amino acids and Internal Standards	Amino acids	MS/MS Transition
Ala<br>**$^{2}H_{3}$-Ala**	Alanine	90.1>44.0<br>93.1>47.1
Arg<br>**$^{2}H_{4}$, ^{13}C-Arg**	Arginine	175.1>70.1<br>180.1>75.1
Asa<br>**$^{2}H_{4}$, ^{13}C-Arg**	Argininosuccinic acid	291.1>70.1<br>180.1>75.1
Cit<br>**2H2-Cit**	Citrulline	176.1>113.1<br>178.1>115.1
Gly<br>**^{15}N,2-^{13}C-Gly**	Glycine	76.0>30.0<br>78.0>32.0
Leu/Ile/Pro-OH<br>**$^{2}H_{3}$-Leu**	Leucine/Isoleucine/Hydroxyproline	132.1>86.1<br>135.1>89.1
Met<br>**$^{2}H_{3}$-Met**	Methionine	150.1>104.1<br>153.1>107.1
Orn<br>**$^{2}H_{6}$-Orn**	Ornithine	133.1>70.1<br>139.1>76.1
Phe<br>**$^{13}C_{6}$-Phe**	Phenylalanine	166.1>120.1<br>172.1>126.1
Tyr	Tyrosine	182.1>136.1

**$^{13}C_6$-Tyr**		188.1>142.1
Val<br><br>**^{15}N,2-$^{13}C_5$-Val**	Valine	118.1>72.1<br><br>124.1>77.1
**Pro**<br><br>**$^{13}C_5$-Pro**	Proline	116.1>70.1<br><br>121.1>74.1
**Gln/Lys**<br><br>**$^{13}C_5$-Gln**	Glutamine/Lysine	147.1>84.0<br><br>152.1>88.1
**Glu**<br><br>**$^{13}C_5$-Gln**	Glutamic acid	148.1>84.0<br><br>152.1>88.1
**Abbreviation Acylcarnitines and Internal Standards**	**Acylcarnitines**	**Transition**
C0<br>**$^2H_9$-C0**	Free Carnitine	162.1>1030<br>171.2>103.0
C2<br>**$^2H_3$-C0**	Acetylcarnitine	204.1>85.0<br>207.1>85.0
C3<br>**$^2H_3$-C3**	Propionylcarnitine	218.1>85.0<br>221.2>85.0
C4<br><br>C3DC/C4OH<br><br>**$^2H_3$-C4**	Butyrylcarnitine<br><br>Malonylcarnitine/3-Hydroxy-butyrylcarnitine	232.2>85.0<br>248.1>85.0<br><br>235.2>85.0
C5<br><br>C5:1<br><br>C4DC/C5OH<br><br>**$^2H_9$-C5**	Valerylcarnitine<br><br>Tiglylcarnitine<br><br>Methylmalonylcarnitine/3-Hydroxy-valerylcarnitine	246.2>85.0<br>244.2>85.0<br>262.1>85.0<br><br>255.2>85.0
C6<br>**$^2H_3$-C6**	Hexanoylcarnitine	260.2>85.0<br>263.2>85.0
C5DC/C6OH<br>C6DC	Glutarylcarnitine/3-Hydroxy-hexanoylcarnitine	276.2>85.0

**$^{2}H_{6}$-C5DC**	Adipylcarnitine	290.2>85.0<br>282.2>85.0
C8:1<br>C8<br>**$^{2}H_{3}$-C8**	Octenoylcarnitine<br>Octanoylcarnitine	286.2>85.0<br>288.2>85.0<br>291.2>85.0
C10<br>C10:2<br>C10:1<br>**$^{2}H_{3}$-C10**	Decanoylcarnitine<br>Decadienoylcarnitine<br>Decenoylcarnitine	316.2>85.0<br>312.2>85.0<br>314.2>85.0<br>319.3>85.0
C12<br>C12:1<br>**$^{2}H_{3}$-C12**	Dodecenoylcarnitine<br>Dodecanoylcarnitine	344.3>85.0<br>342.3>85.0<br>347.3>85.0
C14C14:1<br>C14:2<br>C14OH<br>**$^{2}H_{3}$-C14**	Tetradecanoylcarnitine (myristoylcarnitine)<br>Tetradecenoylcarnitine<br>Tetradecadienoylcarnitine<br>3-Hydroxy-tetradecanoylcarnitine	372.3>85.0<br>370.3>85.0<br>368.3>85.0<br>388.3>85.0<br>375.3>85.0
C16:1	Hexadecenoylcarnitine	398.3>85.0

C16<br><br>C16OH<br><br>**$^{2}H_{3}$-C16**	Hexadecanoylcarnitine (Palmitoylcarnitine)<br><br>3-Hydroxy-hexadecanoylcarnitine	400.3>85.0<br><br>416.3>85.0<br><br>403.4>85.0
C16:1OH/C17<br><br>**$^{2}H_{3}$-C16**	3-Hydroxy-hexadecenoylcarnitine	414.3>85.0<br><br>403.4>85.0
C18<br><br>C18:1<br><br>C18:2<br><br>C18:1OH<br><br>C18OH<br><br>C18:2OH<br><br>**$^{2}H_{3}$-C18**	Octadecanoylcarnitine (Stearoylcarnitine)<br><br>Octadecenoylcarnitine (Oleylcarnitine)<br><br>Octadecadienoylcarnitine (Linoleylcarnitine)<br><br>3-Hydroxy-octadecenoylcarnitine<br><br>3-Hydroxy-octadecanoylcarnitine3-Hydroxy-octadecadienoylcarnitine	428.4>85.0<br><br>426.4>85.0<br><br>424.3>85.0<br><br>442.4>85.0<br><br>444.4>85.0<br><br>440.3>85.0<br><br>431.4>85.2
C20<br><br>**$^{2}H_{3}$-C26**	Eicosanoylcarnitine (Arachidoylcarnitine)	456.4>85.0<br><br>543.5>85.0
C22<br><br>**$^{2}H_{3}$-C26**	Docosanoylcarnitine (Behenoylcarnitine)	484.4>85.0<br><br>543.5>85.0
C24<br><br>**$^{2}H_{3}$-C26**	Tetracosanoylcarnitine (Lignoceroylcarnitine)	512.5>85.0<br><br>543.5>85.0
C26<br><br>**$^{2}H_{3}$-C26**	Hexacosanoylcarnitine (Cerotoylcarnitine)	540.5>85.0<br><br>543.5>85.0
**Abbreviation Ketones<br><br>and Internal Standards**	**Ketones**	**Transition**
SA	Succinylacetone	155.1>109.1

**$^{13}C_5$-MPP[2]**		160.1>114.1
**Abbreviation Nucleosides and Internal Standards**	**Nucleosides**	**Transition**
ADO<br>**$^{13}C_5$-ADO**	Adenosine	268.1>136.1<br>273.1>136.1
D-ADO<br>**$^{13}C_5$-dADO**	2'-deoxyadenosine	252.1>136.1<br>257.1>136.1
**AbbreviationLysophospholipids and Internal Standards**	**Lysophospholipids**	**Transition**
C20:0-LPC<br>**$^{2}H_4$-C26:0-LPC**	C20:0 lysophosphatidylcholine	552.4>104.1<br>640.5>104.1
C22:0-LPC<br>**$^{2}H_4$-C26:0-LPC**	C22:0 lysophosphatidylcholine	580.4>104.1<br>640.5>104.1
C24:0-LPC<br>**$^{2}H_4$-C26:0-LPC**	C24:0 lysophosphatidylcholine	608.5>104.1<br>640.5>104.1
C26:0-LPC<br>**$^{2}H_4$-C26:0-LPC**	C26:0 lysophosphatidylcholine	636.5>104.1<br>640.5>104.1

Table S2: Plasma quantitative levels of metabolites expressed as μM and metabolite ratios measured in all samples**.**

Table S3: Plasma levels of Amino acids and Acylcarnitine quantified in the comparison between transient loss of consciousness (TLC) patients and control (CTRL) groups, described in term of mean, standard deviation (SD), p-value and False Discovery Rate (FDR).

Compound Name	Mean in TLC (μM)	SD in TLC	Mean in CTRL (μM)	SD in CTRL	p.value	FDR
ORN	134.25	42.14	90.26	16.98	0.002	0.076
VAL	365.34	71.37	291.22	58.92	0.002	0.076
LEU-ILE-PRO-OH	318.59	71.37	244.62	59.14	0.002	0.076
PRO	280.19	92.98	203.69	38.05	0.013	0.230
MET/PHE	0.38	0.07	0.44	0.07	0.013	0.230
C18	0.05	0.02	0.03	0.01	0.019	0.230
C18:2	0.06	0.03	0.04	0.01	0.023	0.230
LEU/ALA	0.59	0.13	0.49	0.12	0.024	0.230
GLU	137.43	71.76	85.26	27.10	0.028	0.230
C0/(C16+C18)	334.95	107.74	415.98	124.35	0.028	0.230
C18:1	0.20	0.09	0.14	0.03	0.032	0.230
ARG	157.82	39.21	129.68	31.46	0.033	0.230
C5:1	0.01	0.01	0.01	0.00	0.035	0.230
MET	43.85	9.19	37.56	7.19	0.042	0.253

Table S4: Plasma levels of Amino acids and Acylcarnitine quantified in the comparison between cardiac syncope (CS) and control (CTRL) groups, described in term of p-value, -log10(p) and False Discovery Rate (FDR).

Name	p.value	-log10(p)	FDR
**PHE**	3.4273E-4	3.465	0.0095965
**PHE/TYR**	2.4887E-4	3.604	0.0095965
**ARG**	9.3449E-4	3.0294	0.015699
**CIT**	0.0021841	2.6607	0.026209
**ORN**	7.8959E-4	3.1026	0.015699
**C18**	2.0542E-4	3.6874	0.0095965
**C18OH**	0.0013317	2.8756	0.018644

Table S5: Plasma levels of Amino acids and Acylcarnitine quantified in the comparison between orthostatic hypotension (OH) and control (CTRL) groups, described in term of p-value, -log10(p) and False Discovery Rate (FDR).

**Name**	**p.value**	**-log10(p)**	**FDR**
PHE	2.0299E-4	3.6925	0.017051
PRO	0.0023113	2.6361	0.097074

Table S6: Plasma levels of Amino acids and Acylcarnitine quantified in the comparison between unexplained syncope (US) and control (CTRL) groups, described in term of p-value, -log10(p) and False Discovery Rate (FDR).

Name	p.value	-log10(p)	FDR
PHE	0.0011207	2.9505	0.047068
PHE/TYR	9.0159E-4	3.045	0.047068

Table S7: Plasma levels of Amino acids and Acylcarnitine quantified in the comparison between NMS and control (CTRL) groups, described in term of p-value, -log10(p) and False Discovery Rate (FDR).

Name	p.value	-log10(p)	FDR
LEU-ILE-PRO-OH	0.0016453	2.7837	0.097515
VAL	0.0023218	2.6342	0.097515

Table S8: Logistic Regression algorithm details based on plasma levels of C24 and C22:0-LPC calculated in orthostatic hypotension (OH) and neurologically mediated syncope (NMS) groups used to re-classification of unexplained syncope (US) patients.

Logistic Regression Model with C22:00LPC and C24

Logit(P)= log(P/(1-P))= 0.928-0.613 C22:0-LPC – 42.405 C24

Where P is Pr(y=1/x)

The best threshold for the predicted P is 0.5.

Label=NMS/OH

Logistic Regression Model-Summary of Features

	Estimate	STD. Error	Z value	Pr(>|z|)
intercept	0.928	0.605	1.535	0.125
C22:0-LPC	-0.613	0.601	-1.02	0.308
C24	-42.405	33.78	-1.25	0.209

Fig. S1: Plasma levels Acylcarnitine C18OH (A), C10:2 (B), C12:1 (C) and C14OH (D) quantified in the comparison between orthostatic hypotension (OH) and cardiac syncope (CS) groups. *means p-value at test t <0 .05

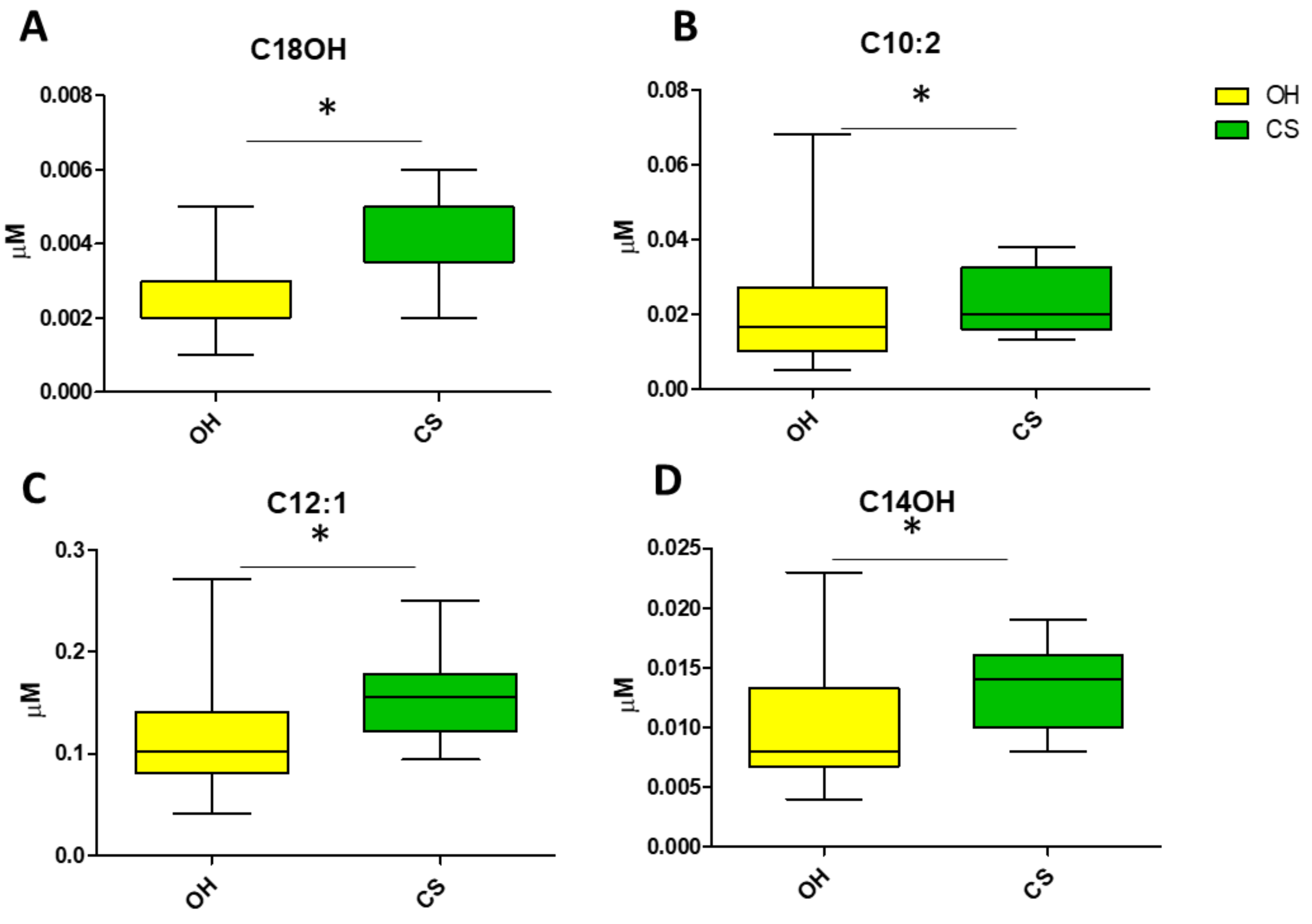

Table S9: Logistic Regression algorithm details based on plasma levels of ASA-Total and ARG calculated in orthostatic hypotension (OH) and cardiac syncope (CS) groups used to re-classification of unexplained syncope (US) patients.

Logistic Regression Model with ARG and ASA-Total

Logit(P)= log(P/(1-P))= 6.989-22.963 ASA-Total – 0.032 ARG

Where P is Pr(y=1/x)

The best threshold for the predicted P is 0.63.

Label=CS/OH

Logistic Regression Model-Summary of Features

	Estimate	STD. Error	Z value	Pr(>|z|)
intercept	6.989	3.213	2.175	0.03
ASA-Total	-22.963	12.382	-1.855	0.06
ARG	-0.032	0.018	-1.813	0.07

Fig. S2: Plasma levels of Acylcarnitine C18OH, C18, C11OH, C10, C4, C14, C24, C12:1 C12 quantified in the comparison between cardiac syncope (CS) and neurologically mediated syncope (NMS) patient groups. *means p-value at test t <0 .05; ** means p-value at test t <0 .01

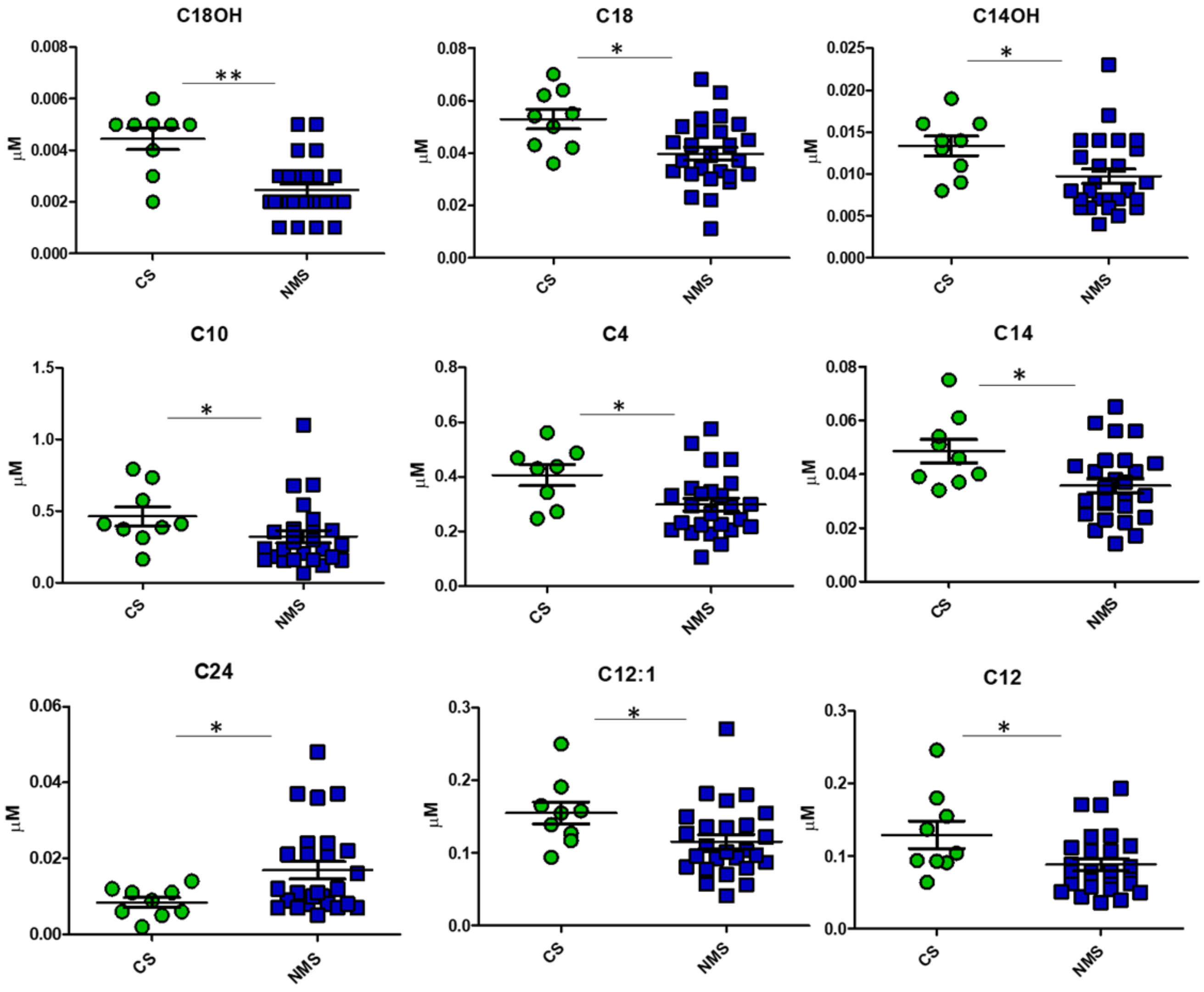

Table S10: Logistic Regression algorithm details based on plasma levels of GLN\LYS calculated in cardiac syncope (CS) and neurologically mediated syncope (NMS) groups used to re-classification of unexplained syncope (US) patients.

Logistic Regression Model with GLN\LYS

Logit(P)= log(P/(1-P))= 6.299-0.003 GLN\LYS

Where P is Pr(y=1/x)

The best threshold for the predicted P is 0.73.

Label=CS/NMS

Logistic Regression Model-Summary of Features

	Estimate	STD. Error	Z value	Pr(>|z|)
intercept	6.299	2.901	2.171	0.03
GLN\LYS	-0.003	0.002	-1.871	0.06

Table S11: Logistic Regression algorithm details based on plasma levels of C22:0-LPC calculated in cardiac syncope (CS) and neurologically mediated syncope (NMS) groups used to re-classification of unexplained syncope (US) patients.

Logistic Regression Model with C22:0-LPC

Logit(P)= log(P/(1-P))= 0.32+2.356 C22:0-LPC

Where P is Pr(y=1/x)

The best threshold for the predicted P is 0.62.

Label=CS/NMS

Logistic Regression Model-Summary of Features

	Estimate	STD. Error	Z value	Pr(>|z|)
intercept	-0.32	0.742	-0.431	0.66
C22:0-LPC	2.356	1.317	1.789	0.07
